# Periodic Visuotactile Stimulation Slowly Enhances the Rubber Hand Illusion in Individuals with High Autistic Traits

**DOI:** 10.3389/fnint.2016.00021

**Published:** 2016-06-09

**Authors:** Masakazu Ide, Makoto Wada

**Affiliations:** ^1^Developmental Disorders Section, Department of Rehabilitation for Brain Functions, Research Institute of National Rehabilitation Center for Persons with DisabilitiesSaitama, Japan; ^2^Japan Society for the Promotion of ScienceTokyo, Japan

**Keywords:** stimulus periodicity, rubber hand illusion, autistic traits, interoceptive sensation, visuotactile integration, body representation

## Abstract

In a rubber hand illusion (RHI) task, synchronous brush stroking of a rubber hand and a participant's hidden hand induces body ownership of the rubber hand. The effects of spatial distances and temporal lags on the RHI have been extensively examined; however, the effect of periodicity of the stimuli on illusory body ownership has not been examined. Meanwhile, the occurrence of RHI tends to be weak in individuals with autism-spectrum disorders (ASD) and high autistic traits. Preference for stimulus having regularity of tempo is generally observed in individuals with ASD, and thus, periodic stimulation might be more effective to elicit the body ownership illusion in individuals with high autistic traits. Hence, we investigated whether stimulus periodicity influenced RHI as well as its association with participant's autistic traits. Brush strokes were applied to a participant's own hand and the rubber hand periodically (2 s) or non-periodically (1–3 s), either synchronously or asynchronously. Two blocks were performed in each condition. We found that periodic stimulation enhanced the spatial updating of tactile sensation induced by RHI in the subsequent block in participants with high autistic traits, whereas both periodic and non-periodic stimulation strongly elicited RHI in blocks 1 and 2. These results indicate that the periodicity of stimulation has different effects based on an individual's autistic traits. Since individuals with ASD are known to sustain their focus on interoceptive sensations (heartbeats), a periodic stimulation that is potentially correlated with heartbeats might be effective to enhance the visuotactile integration during RHI in individuals with high autistic traits.

## Introduction

In a rubber hand illusion (RHI), a participant's hidden hand and a rubber hand are synchronously stroked by paintbrushes, and an individual gradually feels as if the rubber hand is his or her own hand (Botvinick and Cohen, 1998). Concurrently, participants feel that their own hands are more toward the rubber hand (proprioceptive drift). Spatiotemporal congruency between visual and tactile (visuotactile) stimuli is considered a crucial factor for RHI. Consistent with this notion, the distance between the participant's hand and the rubber hand should be within a certain range on both horizontal (Lloyd, 2007) and vertical axes (Kalckert and Ehrsson, 2014) to induce RHI. In addition, anatomical plausibility of the hand also affects RHI because the illusion occurs only when the joint angles are within ranges in which participants can mimic the posture (Tsakiris and Haggard, 2005; Ide, 2013). Regarding temporal limitations of the illusion, visuotactile stimuli must be presented within a given amount of temporal lag (~300 ms) (Shimada et al., 2009, 2014). Thus, congruent spatiotemporal stimuli can selectively induce strong RHI.

In these previous studies, the effect of the spatial proximity and synchronicity of the stimuli have been examined, but periodicity of the stimulus has been never considered. In previous experiments, manual stimulation was delivered in constant/periodical intervals ~0.5–1 Hz (Ehrsson et al., 2005; Shimada et al., 2009; Suzuki et al., 2013). Since interoceptive sensation (e.g., heartbeats) also has periodicity (~1 Hz), there is a commonality between the periodicity of stimulation to induce RHI and that of heartbeats. Recent studies have suggested that visual feedback of heartbeats on the rubber hand (change in color; Suzuki et al., 2013) or a full body image (flashes; Aspell et al., 2013) induces illusory body ownership. To our knowledge, no study is available that directly compared the effect of stimulus periodicity (periodic or non-periodic) on illusory body ownership. Thus, it is intriguing to examine the effect of periodicity of synchronous stimuli on the illusory sensation during the RHI task, since such a periodic feature of stimulation might play an important role in inducing the ownership of the rubber hand.

Meanwhile, individuals with autism-spectrum disorders (ASD) have several symptoms such as severe and sustained impairment in social interaction, deviance in communication, and restricted interests or repetitive behavior patterns. It is known that they process information from different sensory modalities in atypical styles compared with neurotypical persons (Kanner, 1943; Marco et al., 2011). Recent studies have started to reveal diverse characteristics regarding their sensory processing, especially for multisensory integration and interaction with the external environment. For example, the temporal binding window of audiovisual stimuli is larger in individuals with ASD compared with neurotypical persons (Foss-Feig et al., 2010; Kwakye et al., 2011). Moreover, diversities of illusory body ownership during RHI in individuals with ASD indicate abnormalities in visuotactile integration. In a reach-to-grasp movement subsequent to the induction of RHI, the normalized integrated jerk of reach-to-grasp movements significantly increased in neurotypical persons, whereas execution of the movement was not affected by the illusion in individuals with ASD (Palmer et al., 2015a). Delayed occurrence of the proprioceptive drift during RHI was also reported, such that the drift was not observed until a subsequent block. In contrast, the proprioceptive drift was obvious in the first block in neurotypical persons (Cascio et al., 2012). Similar features were also seen in individuals with high autistic traits without a diagnosis of ASD. They tended to exhibit small proprioceptive drifts during RHI (Paton et al., 2012; Palmer et al., 2013). Our recent study has suggested that subjective feeling of ownership of the rubber hand can be also predicted by individual's autistic traits, especially the degree of social communication ability (Ide and Wada, 2016). Thus, individuals with high autistic traits seem to have common properties to those of individuals with ASD regarding the features of body ownership.

Autistic traits form a wide spectrum, and several such traits can be seen even in nonclinical persons (Palmer et al., 2015b). Thus, in line with the previous study with individuals with ASD (Cascio et al., 2012), we predicted that delayed occurrence of RHI would be observable in individuals with high autistic traits. In this study, we investigated the effect of stimulus periodicity on the RHI. Previously, in individuals with ASD, preference for stimulus with regularity of tempo was observed (Hutt and Hutt, 1968; Frankel et al., 1978; Garretson et al., 1990). We assumed that this preference for regularity of an external stimulus might reflect their enhanced ability to sustain their own interoceptive sensation, because it has been reported that external stimulus which is synchronously delivered with participants' own heartbeats selectively attracts their attention (Kroeze and van den Hout, 2000; Pineles and Mineka, 2005). Thus, we hypothesized that periodic stimulation might be more effective to elicit illusory body ownership in individuals with high autistic traits than with low autistic traits. In the present study, we investigated whether periodic stimulation dominantly enhanced the illusory body ownership in nonclinical individuals with high autistic traits using PC-controlled machinery stimulators that can easily control the timing of the stimulus.

## Materials and methods

### Participants

Twenty-two participants (18 females, 4 males; 23 ± 2.9 years old) participated in this study after we excluded two participants who continuously failed to perform the proprioceptive drift task. All participants had normal or corrected-to-normal (e.g., glasses, contacts) vision and no history of neurological diseases. We scored their handedness according to the Edinburgh Inventory (Oldfield, 1971). In total, 19 participants were right-handed (+50 < *L.Q*. < + 100, 84.7 ± 15.8), two participants were ambidextrous (*L.Q*. = 10, 0, respectively), and one participant was left-handed (*L.Q*. = −30). Additionally, we assessed the participants' autistic traits using the Japanese version of the Autism Spectrum Quotient (AQ) score (Wakabayashi et al., 2004), which was translated from the English version (Baron-Cohen et al., 2001). The mean AQ score of the participants was 17.3 ± 3.63 (mean ± *SD*). The ethics committee of the National Rehabilitation Center for Persons with Disabilities approved the study; informed consent was obtained from all participants before the experiments.

### Stimulus

**Figure 1:**
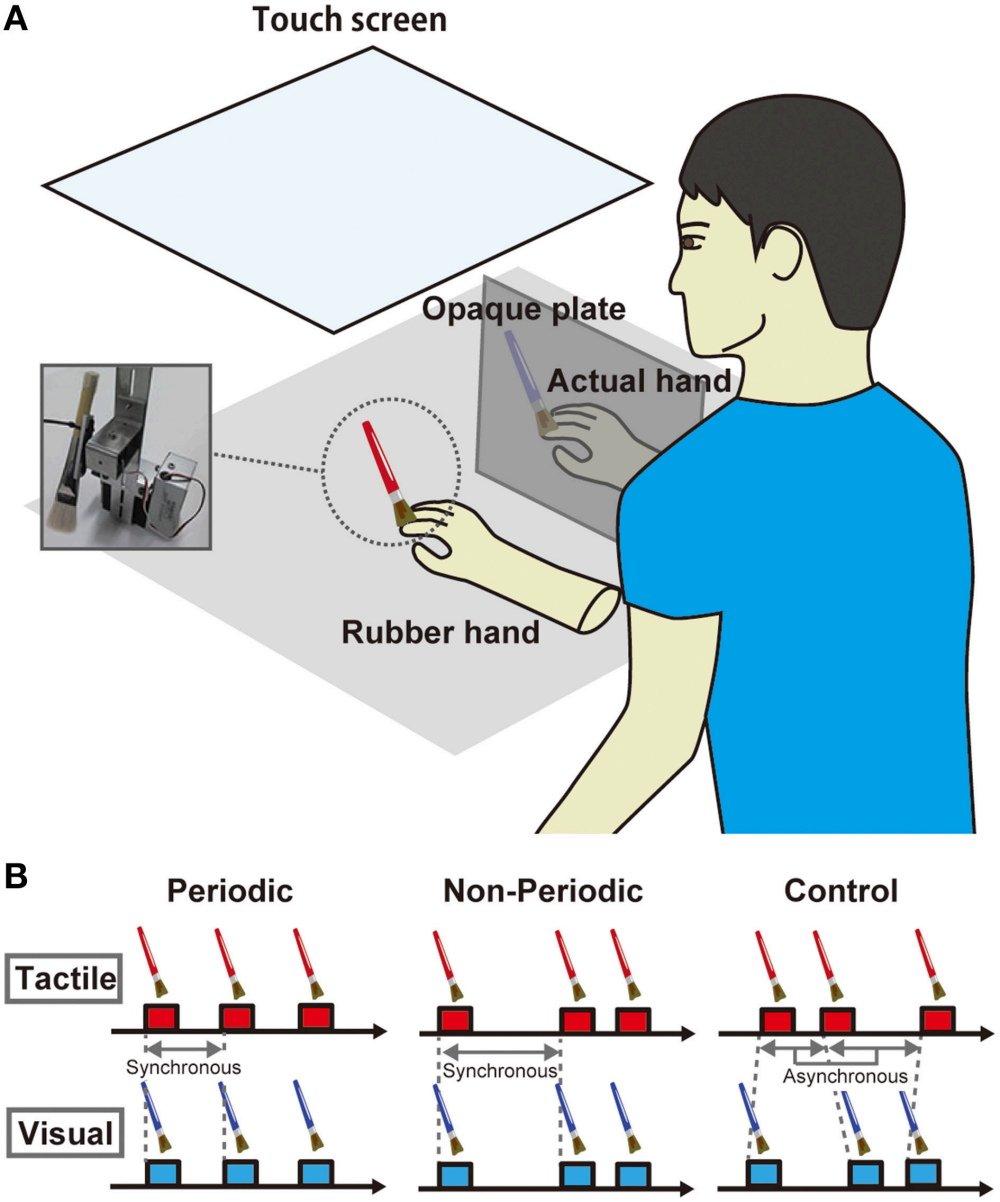
**(A)** Task setting. A rubber hand was placed on the desk in front of the participants. Brush stroking was delivered to the rubber hand and the participant's own hand using PC-controlled machinery stimulators. A touch screen was placed above the rubber hand and the participant's hand while the participant could see the rubber hand. **(B)** Task conditions. There were three conditions in terms of the temporal parameters of stimulus periodicity. In the *periodic condition*, the intervals of synchronous brush stroking to the rubber and actual hands were constant at 2 s. In the *non-periodic condition*, the intervals were randomly assigned and ranged from 1 to 3 s. In the *asynchronous condition*, the intervals were individually and randomly assigned to the rubber hand and actual hand and ranged from 1 to 3 s.

A rubber hand (Otto Bock, Duderstadt, Germany) was placed on the desk in front of the participant (Figure 1). For brush stroking, we used a customized device with two brushes that were attached on DC motors (MS025, Solidray Co Ltd, Japan); the timings of the stimuli were arbitrarily assigned by a personal computer (VAIO, SONY, Japan) with Matlab software (MathWorks, Natick, MA, USA). Strokes from the reciprocating brushes were synchronously delivered to the rubber hand and the participant's own hand at a velocity of maximum 545.5°/s (80 cm/s). A touch screen (KTMT-1921 Add-On Touch Screen for 20–21″ LCD & CRT, KEYTEC, Inc., Garland, TX, USA) was placed above the rubber hand and the participant's hand while the participant could see the rubber hand. The participants wore earplugs and noise-canceling headphones to block sounds generated by the motor.

### Procedure

The rubber hand was placed in front of the participant while her/his right hand was placed on the right side of the rubber hand (13 cm from the rubber hand); the right hand was hidden from view by an opaque plate. We asked the participant to fixate on an index finger of the rubber hand where the brush strokes were delivered. We adopted three conditions regarding the temporal parameters of the visuotactile stimulation (Figure 1B). In the *periodic condition*, intervals of synchronous brush stroking on the rubber and actual hands were constant at 2 s. In the *non-periodic condition*, the intervals were randomly assigned within 1 to 3 s, while the total numbers of stimuli were the same as that in the periodic condition, and the mean interval in the non-periodic condition was ~2 s. In *the asynchronous condition*, the intervals were individually and randomly assigned to the rubber hand and the actual hand within a range of 1 to 3 s. There were six stimulation blocks in total, so we performed each condition twice. One block requires 3 min, and the entire experiment, including rest periods between blocks, was performed in 30 min.

We asked the participants to perform two tasks to measure the degree of RHI. First, the participants answered the degree of their subjective feeling of RHI in questionnaires that were translated into Japanese Likert 7-point scale from +3 (agree strongly) to −3 (disagree strongly; Table 1). In the questionnaires, we assumed that only three of nine items relate to the subjective feeling of RHI (Botvinick and Cohen, 1998). Second, the participants were required to use their left hand to point at the place on the touch screen where they felt their right index finger. We calculated the amount of proprioceptive drift by subtracting the horizontal position indicated prior to the block from the position indicated after the block.

**Table 1:** **Questionnaires for subjective feeling of RHI (Botvinick and Cohen, 1998)**.

**Items**	**Text**
Item 1	It seemed as if I were feeling the touch of the paintbrush in the location where I saw the rubber hand touched.
	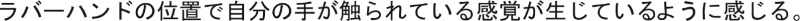
Item 2	It seemed as though the touch I felt was caused by the paintbrush touching the rubber hand.
	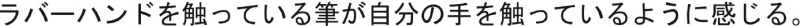
Item 3	I felt as if the rubber hand were my hand.
	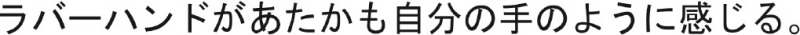
Item 4	I felt as if my hand were drifting toward the rubber hand.
	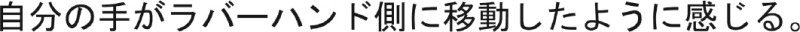
Item 5	It seemed as if I might have more than one left hand.
	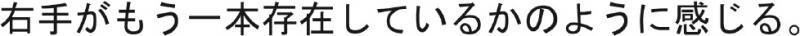
Item 6	It seemed as if the touch I was feeling came from somewhere between my own hand and the rubber hand.
	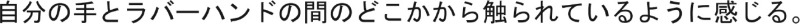
Item 7	I felt as if my hand were turning rubbery.
	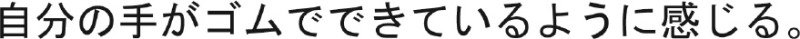
Item 8	It appeared as if the rubber hand were drifting toward my hand.
	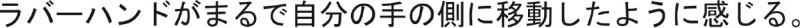
Item 9	The rubber hand began to resemble my own hand, in terms of shape, skin tone, freckles, or some other visual feature.
	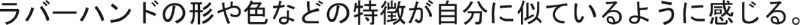

*We assumed that only three of nine items in the questionnaire are related to the subjective feeling of RHI. The questionnaire was translated into Japanese*.

Table 2 indicates mean scores of subjective feeling of RHI that was assessed by the three questionnaires and proprioceptive drifts in each block of each stimulus condition. We defined *change in subjective feeling of RHI* (Sync–Async) by subtracting individual scores in items 1, 2, and 3 on the questionnaires (see Table 1) in the asynchronous condition from those scores in synchronous conditions (periodical and non-periodical conditions). We averaged the scores from two blocks of each condition. Similarly, we calculated *change in the proprioceptive drift* (Sync–Async) by subtracting the amount of proprioceptive drift in the asynchronous condition from the amount in the synchronous conditions.

**Table 2:** **Mean scores (±SEM) for questionnaires relating the subjective feeling of RHI (item 1, 2, and 3) and proprioceptive drift**.

	**Item 1**		**Item 2**		**Item 3**		**Proprioceptive drift**	
	**Block 1**	**Block 2**	**Block 1**	**Block 2**	**Block 1**	**Block 2**	**Block 1**	**Block 2**
Periodic	1.68 (0.36)	1.59 (0.39)	1.86 (0.25)	2.05 (0.26)	1.86 (0.27)	1.81 (0.18)	1.81 (0.87)	3.02 (0.79)
Non-Periodic	1.59 (0.35)	0.95 (0.86)	2.14 (0.23)	1.64 (0.31)	1.95 (0.25)	1.55 (0.28)	1.73 (0.81)	3.41 (0.92)
Asynchronous	−0.77 (0.47)	−0.73 (0.49)	−0.82 (0.39)	−0.82 (0.39)	−0.55 (0.45)	−0.5 (0.31)	0.11 (0.88)	1.41 (0.51)

## Results

In the behavioral test, most participants (21 out of the 22 participants) reported that they felt RHI during both periodic and non-periodic (but synchronous) stimulations (Table 2). Most participants felt stronger subjective feeling of RHI as indicated by at least one of the three items in synchronous conditions (periodical and non-periodical) compared with asynchronous condition.

### Effect of periodic stimulation on RHI in the entire group

**Figure 2:**
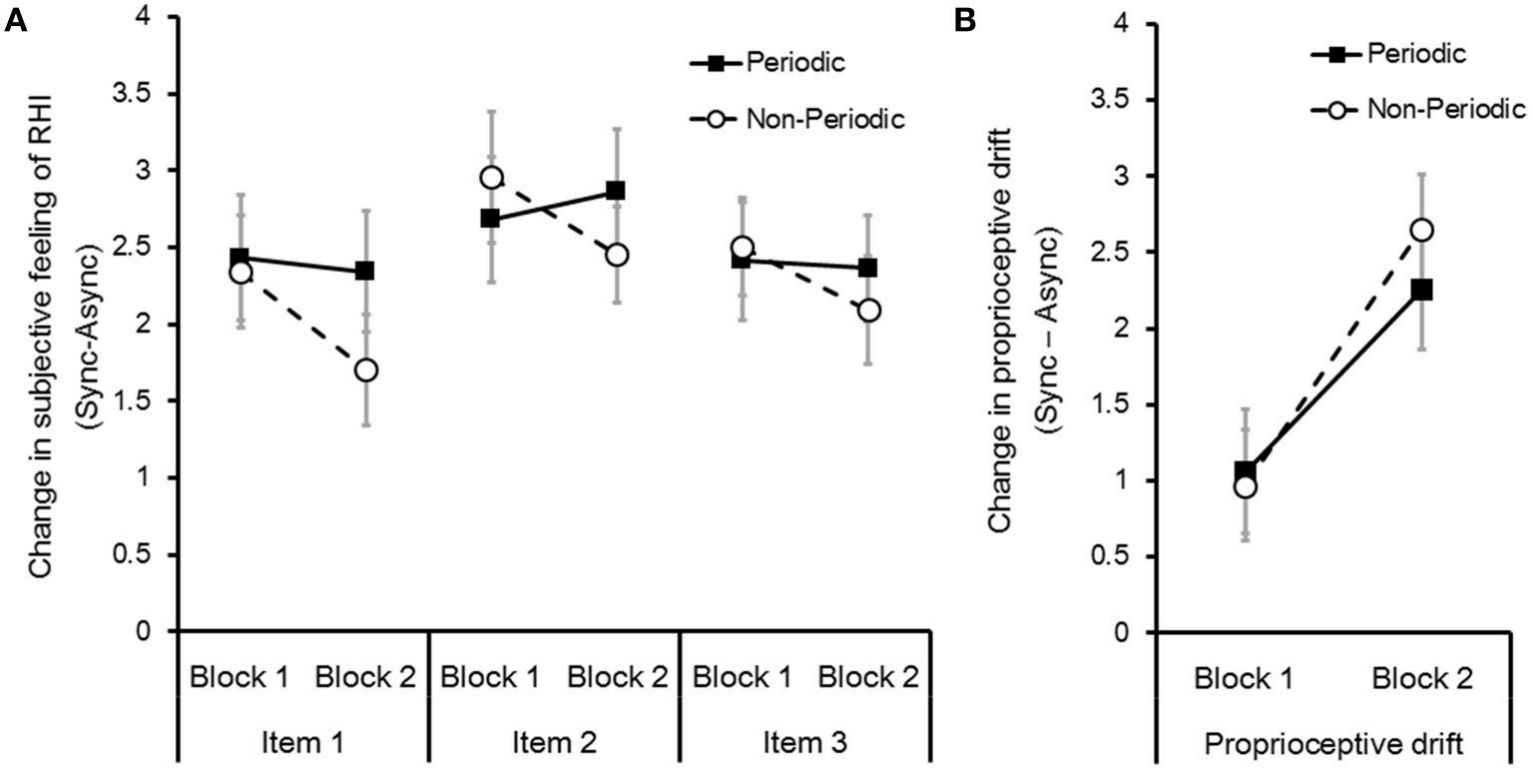
**(A)** Effect of stimulus periodicity on the *change in subjective feeling of RHI* (Sync–Async). *Change in subjective feeling of RHI* were calculated for items 1, 2, and 3 in the questionnaire (see Table 1) and blocks 1 and 2. Error bars denote the standard error of the mean (*N* = 22). **(B)** Effect of the stimulus periodicity on *change in the proprioceptive drift* (Sync–Async).

To examine the effects of stimulus periodicity on RHI, we conducted two-way repeated measures ANOVA with stimulus periodicities (periodical or non-periodical) and blocks (first or second) in the *change in subjective feeling of RHI* (item 1, 2, and 3, respectively) of the entire group (*N* = 22). We did not identify a significant main effect for all items of the periodical conditions [*Fs*_(1, 21)_ = 2.54, 0.16, and 0.23; *ps* = 0.13, 0.69, and 0.64; partial η^2^ = 0.11, 0.01, 0.01; items 1, 2, and 3, respectively] and blocks [*Fs*_(1, 21)_ = 2.34, 0.7, and 1.42; *ps* = 0.14, 0.41, and 0.25; partial η^2^ = 0.1, 0.03, 0.07; items 1, 2, and 3, respectively] (Figure 2A) based on the defined criteria (Cohen, 1988; Ellis, 2010). We also did not identify any significant interactions among periodical conditions and blocks in all items [*Fs*_(1, 21)_ = 2.78, 1.49, and 3.65; *ps* = 0.11, 0.24, and 0.69; partial η^2^ = 0.12, 0.15, 0.07; items 1, 2, and 3, respectively].

We also conducted two-way repeated measures ANOVA with stimulus periodicity types (periodical or non-periodical) and blocks (first or second) in *change in the proprioceptive drift*. We found that participants started to perceive their own right hand's position to be near the rubber hand in the second block compared with the first block [*F*_(1, 21)_ = 6.41, *p* < 0.05; partial η^2^ = 0.23] (Figure 2B). However, we did not identify any significant effect of periodical conditions [*F*_(2, 42)_ = 2.61, *p* = 0.09; partial η^2^ = 0.005] or an interaction between periodical conditions and blocks [*F*_(1, 21)_ = 0.17, *p* = 0.68; partial η^2^ = 0.01].

### Influence of a participant's autistic traits on the temporal transition of RHI

**Figure 3:**
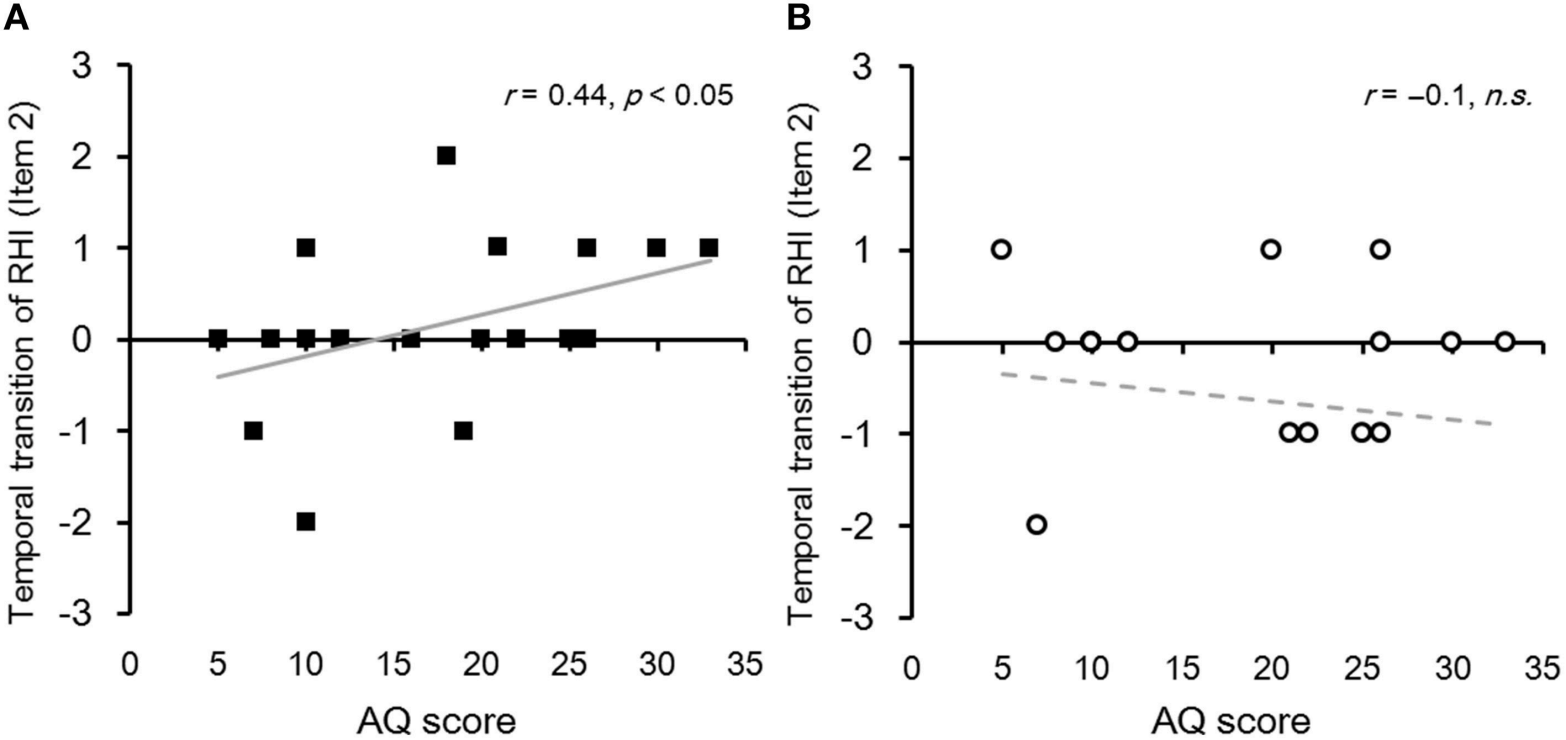
**(A)** Relationship between the temporal transition of RHI and the participant's autistic traits in the periodic condition as assessed by item 2. Each black square indicates each participant's score regarding the temporal transition of *change in subjective feelings of RHI*. A sloped solid gray line indicates a regression linear function fitted with the least-squares method (*N* = 22). **(B)** Relationship between the temporal transition of RHI and the participant's autistic traits in the non-periodic condition assessed by item 2. Each open circle indicates each participant's score for temporal transition of change in subjective feelings of RHI. A sloped dotted gray line indicates a regression linear function fitted with the least-squares method (*N* = 22).

We examined whether the temporal transition of subjective feeling of RHI and proprioceptive drift correlated with each participant's autistic traits. To estimate the *temporal transition of RHI*, we subtracted an individual's scores regarding the change in subjective feeling of RHI in the first block of each stimulus periodicity (periodic/non-periodic) from the scores in the second block (Block 2–1). We found that the AQ score positively correlated with the magnitude of the *temporal transition of RHI* in item 2 in periodic condition (*r* = 0.44, *p* < 0.05) but not in non-periodic condition (Figure 3). No significant correlation was observed for items 1 and 3 in periodic and non-periodic conditions. Regarding the proprioceptive drift, we also subtracted the *change in proprioceptive drift* in the first block from that in the second block (*temporal transition of proprioceptive drift* [Block 2–1]). We did not identify any correlation between the AQ score and the amount of temporal transition in proprioceptive drift for items 1, 2, and 3 (*rs* = −0.06, −0.2, 0.22, and *ps* = 0.78, 0.36, 0.31, respectively).

**Figure 4:**
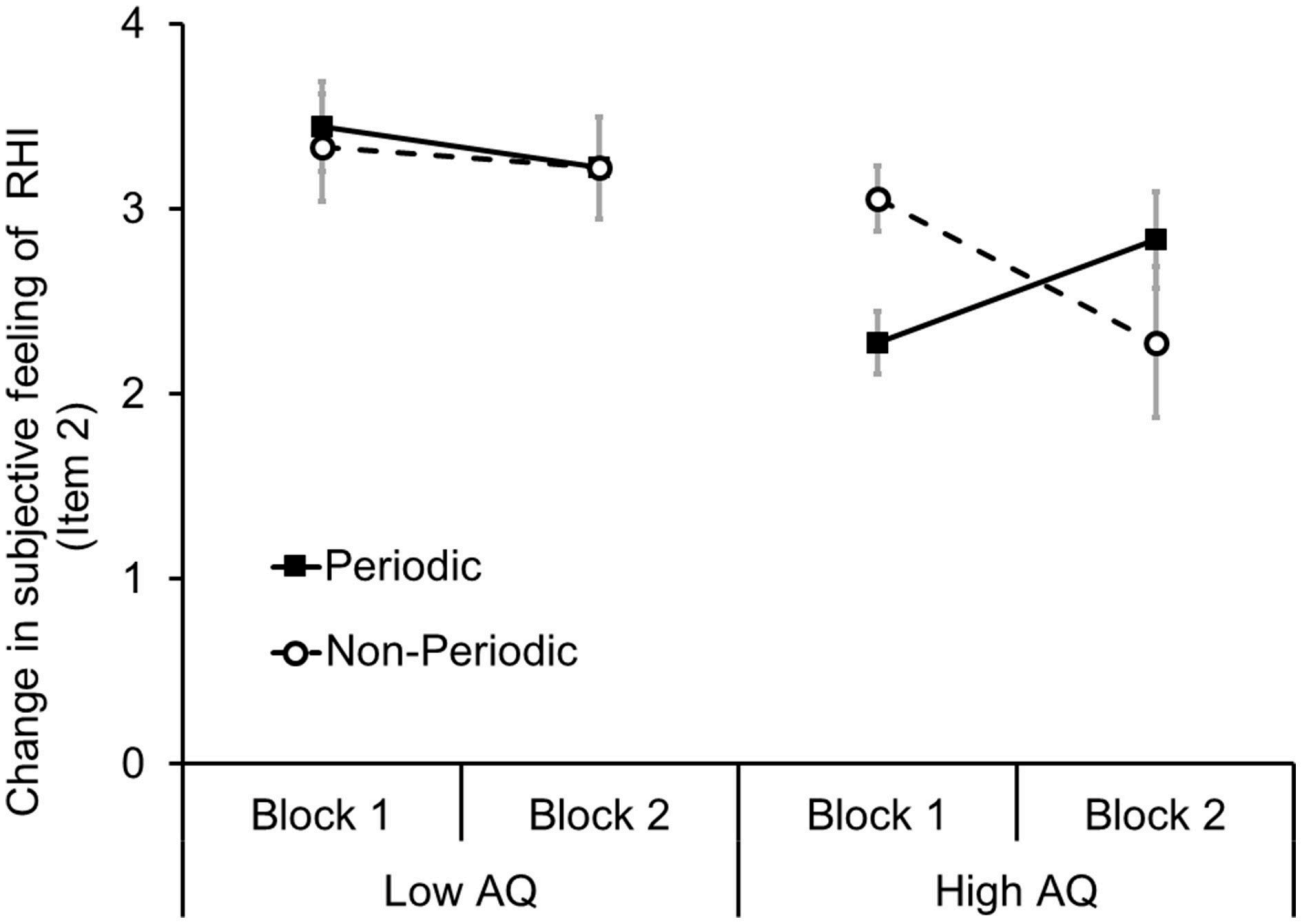
**Effect of stimulus periodicity on spatial updating of tactile sensation (item 2) of RHI in the low AQ and high AQ groups**. A solid line with black squares indicates the mean of each participant's subjective feeling of RHI in the periodic condition, and a dotted line with white circle indicates the mean in the non-periodic condition. Error bars denote the standard error of the mean.

These results, from all participants (*N* = 22), indicated that the subjective feeling of spatial updating of tactile sensation (item 2) gradually increased in individuals with high autistic traits. Thus, we decided to further analyse item 2 to confirm whether the participant's autistic traits influenced the temporal transition of subjective feeling of RHI. To investigate the influence of autistic traits, we divided all participants (*N* = 22) into two groups based on the AQ scores (range: 5–33, *M* = 17.82, *SD* = 3.63). Nine participants with low AQ scores were classified as the low AQ group (range: 5–12, *M* = 10.73, *SD* = 3.7), and nine participants with high AQ scores were classified as the high AQ group (range: 21–33, *M* = 22.1, *SD* = 3.01). Four participants were excluded from this analysis because they had average AQ scores (range: 16–20, *M* = 17, *SD* = 1.71). A three-way one repeated measures ANOVA among groups (high AQ/low AQ), stimulus periodicities (periodical/non-periodical), and blocks (first/second) exhibited a significant interaction effect [*F*_(1, 16)_ = 6.04, *p* < 0.05] (Figure 4). Effect size of the interaction was small (partial η^2^ = 0.27). A *post-hoc* test (Ryan, *p* < 0.05) revealed that the subjective feeling of RHI in the periodical condition in the high AQ group increased in block two in contrast to the non-periodical condition (Cohen's *d* = 1.25). However, in the low AQ group, stimulus periodicity did not have any effect on the subjective feeling of RHI in both blocks 1 and 2. In addition, we did not identify such a tendency in items 1 and 3.

## Discussion

In the present results, periodic visuotactile stimulation gradually promoted the spatial updating of tactile sensation in individuals with high autistic traits. In contrast, difficulty in feeling the illusory body ownership during RHI in individuals with ASD has been previously reported. Proprioceptive drift during RHI was not observed until a subsequent block in such participants (Cascio et al., 2012). Similar features were also seen in individuals with high autistic traits without a diagnosis of ASD. They tended to show small proprioceptive drift during RHI (Paton et al., 2012; Palmer et al., 2013). Current results showed that periodic stimulation could gradually enhance spatial updating of tactile sensation by RHI in individuals with high autistic traits.

Previous reports have demonstrated that visual feedback along with interoceptive information affects the induction of illusory body ownership (Aspell et al., 2013; Suzuki et al., 2013). It has also reported that the accuracy for interoceptive information (e.g., heartbeats) negatively correlates with the degree of illusory body ownership (Tsakiris et al., 2011). Individuals with ASD can sustain interoceptive awareness of their own heartbeats compared with neurotypical persons, and such an increased sustaining time is associated with the degree of RHI (Schauder et al., 2015). It is possible that the ability to focus on one's own internal bodily signals and external signals might exhibit a trade-off relationship. In this context, individuals with ASD (or high autistic traits) might also persist with their focus on an external object (e.g., the rubber hand), if the stimuli having similar periodicity to their own interoception (e.g., heartbeats) were provided. In the present study, periodic stimulation (0.5 Hz) was not presented in completely consistent way to participant's heartbeats, but it would be partly correlated with heartbeats' periodicity (~1 Hz). We speculate that periodical stimulation could gradually facilitate spatial updating of tactile sensation in individuals with high autistic traits, though these participants are known to have difficulty in the occurrence of RHI (Paton et al., 2012; Palmer et al., 2013; Ide and Wada, 2016). To confirm this hypothesis, further experiments using brush stroking with timings of the participant's own heartbeats are needed. In the present study, in both periodic and non-periodic stimulation conditions, synchronous brush stroking elicited the subjective feeling of RHI compared with asynchronous brush stroking. However, with regard to item 2 in the questionnaire, we found that periodic stimulation enhanced the subjective feeling of RHI more in block 2 in individuals with high autistic traits. In item 2 (“It seemed as though the touch I felt was caused by the paintbrush touching the rubber hand.”), it could be presumed that this sensation involved a degree of spatial updating of the tactile sensation from their actual hand to the rubber hand rather than illusory body ownership of the rubber hand. On the other hand, we did not observe such tendency in the spatial change of the feeling (item 1) and illusory ownership of the rubber hand (item 3).

In summary, the current results demonstrate that periodic brush stroking enhances the spatial updating induced by RHI in a delayed fashion in individuals with high autistic traits. These effects may be derived from their sustained awareness of interoceptive sensation (i.e., heartbeats). However, since the results are confined to the subjective feeling of RHI, especially about spatial updating of the rubber hand, this is not applicable to other aspects of illusory body ownership sensation and proprioceptive drift. Thus, further studies are needed to reveal the mechanism by which such strong awareness of interoceptive sensation in individuals with ASD could influence their cognition of external environments that have periodic or non-periodic features in daily life.

## Author contributions

MI conducted the experiments; MW developed the apparatus; MW and MI designed the experiments and wrote the paper.

### Conflict of interest statement

The authors declare that the research was conducted in the absence of any commercial or financial relationships that could be construed as a potential conflict of interest.
